# Flow-dependent regulation of endothelial Tie2 by GATA3 in vivo

**DOI:** 10.1186/s40635-021-00402-x

**Published:** 2021-08-02

**Authors:** Temitayo O. Idowu, Valerie Etzrodt, Thorben Pape, Joerg Heineke, Klaus Stahl, Hermann Haller, Sascha David

**Affiliations:** 1 Department of Nephrology and Hypertension, Hannover Medical School, Hannover, Germany; 2 Department of Cardiovascular Physiology, European Center for Angioscience, Medical Faculty Mannheim, Heidelberg University, Mannheim, Germany; 3 German Center for Cardiovascular Research (DZHK), Partner Site Heidelberg, Mannheim, Germany; 4 Department of Gastroenterology, Hepatology and Endocrinology, Hannover Medical School, Hannover, Germany; 5 Institute of Intensive Care Medicine, University Hospital Zurich, Rämistrasse 100, 8091 Zurich, Switzerland

**Keywords:** Vascular leakage, Permeability, Hypotension, Shock, Blood flow, Capillary leakage

## Abstract

**Background:**

Reduced endothelial Tie2 expression occurs in diverse experimental models of critical illness, and experimental Tie2 suppression is sufficient to increase spontaneous vascular permeability. Looking for a common denominator among different critical illnesses that could drive the same Tie2 suppressive (thereby leak inducing) phenotype, we identified “circulatory shock” as a shared feature and postulated a flow-dependency of Tie2 gene expression in a GATA3 dependent manner. Here, we analyzed if this mechanism of flow-regulation of gene expression exists in vivo in the absence of inflammation.

**Results:**

To experimentally mimic a shock-like situation, we developed a murine model of clonidine-induced hypotension by targeting a reduced mean arterial pressure (MAP) of approximately 50% over 4 h. We found that hypotension-induced reduction of flow in the absence of confounding disease factors (i.e., inflammation, injury, among others) is sufficient to suppress GATA3 and Tie2 transcription. Conditional endothelial-specific GATA3 knockdown (B6-Gata3^tm1-Jfz^ VE-Cadherin(PAC)-cerERT2) led to baseline Tie2 suppression inducing spontaneous vascular leak. On the contrary, the transient overexpression of GATA3 in the pulmonary endothelium (jet-PEI plasmid delivery platform) was sufficient to increase Tie2 at baseline and completely block its hypotension-induced acute drop. On the functional level, the Tie2 protection by GATA3 overexpression abrogated the development of pulmonary capillary leakage.

**Conclusions:**

The data suggest that the GATA3–Tie2 signaling pathway might play a pivotal role in controlling vascular barrier function and that it is affected in diverse critical illnesses with shock as a consequence of a flow-regulated gene response. Targeting this novel mechanism might offer therapeutic opportunities to treat vascular leakage of diverse etiologies.

**Supplementary Information:**

The online version contains supplementary material available at 10.1186/s40635-021-00402-x.

## Background

Vascular permeability is a hallmark of the pathological host response to an infection that also plays a key pathophysiological role in various entities leading to critical illness [1, 2]. Therapeutic strategies that target this hyperpermeability of the endothelium are highly desirable. The endothelial tyrosine kinase receptor Tie2 regulates vascular quiescence and the endothelial response to injury [3–5]. Pulmonary Tie2 expression is rapidly suppressed in many experimental models of critical illness, such as sepsis, hemorrhagic shock, anthrax, malaria and even mesenteric ischemia [6–8]. Looking for a common denominator that could explain this acute drop in Tie2 transcription (90% in just 4 h), it becomes evident that all these disease models share a certain degree of hemodynamic compromise, i.e., shock. Given that some endothelial genes are on the transcriptional level flow-regulated, we hypothesized that shock and accompanied decreased microvascular flow could count for acute changes in Tie2 transcription and associated vascular leak. In fact, van Meurs et al. have proposed such a mechanism in the context of hemorrhagic shock [5, 9].

Endothelial cells (ECs) can sense the reduction in blood flow through a series of complex mechanosensory signaling systems [10–12], among which are flow responsive transcription factors, such as KLF2, RUNX1, GATA4, NF-κB, among others [13, 14]. Low flow is known to alter the endothelium's physiological processes leading to increased vascular permeability and inflammation [15]; however, the mechanisms involved are not fully understood.

To identify potential flow-dependent mediators of Tie2, we screened previously reported Tie2 transcription regulators for their flow-dependent tendencies. By doing so, we identified the transcription factor GATA3 as a potential candidate in ECs in vitro*.* Furthermore, experimental reduction of GATA3 in ECs using siRNA was sufficient to attenuate Tie2 mRNA expression in addition to basal barrier function in vitro [8]. Along these lines, Song et al. [16] have also shown that GATA3 can bind and activate the Tie2 promoter. To our knowledge, it has never been addressed whether this observation of a flow-dependent Tie2 regulation holds true under clean in vivo conditions (aside from disease models, such as sepsis that have strong inflammatory components).

Therefore, we set out to test the role of (1) flow on endothelial Tie2 regulation in a novel in vivo model of hypotension independent of inflammation and (2) the transcription factor GATA3 and its relation to Tie2 in vivo in wild type, knockout and transgenic mice. Our study may provide novel insights into the effect of reduced blood flow on endothelial barrier function during critical illnesses.

## Materials and methods

We adhered to the essential 10 of the ARRIVE guidelines (www.arriveguidelines.org) throughout this study.

### Clonidine induced model of controlled hypotension

**Fig. 1.**
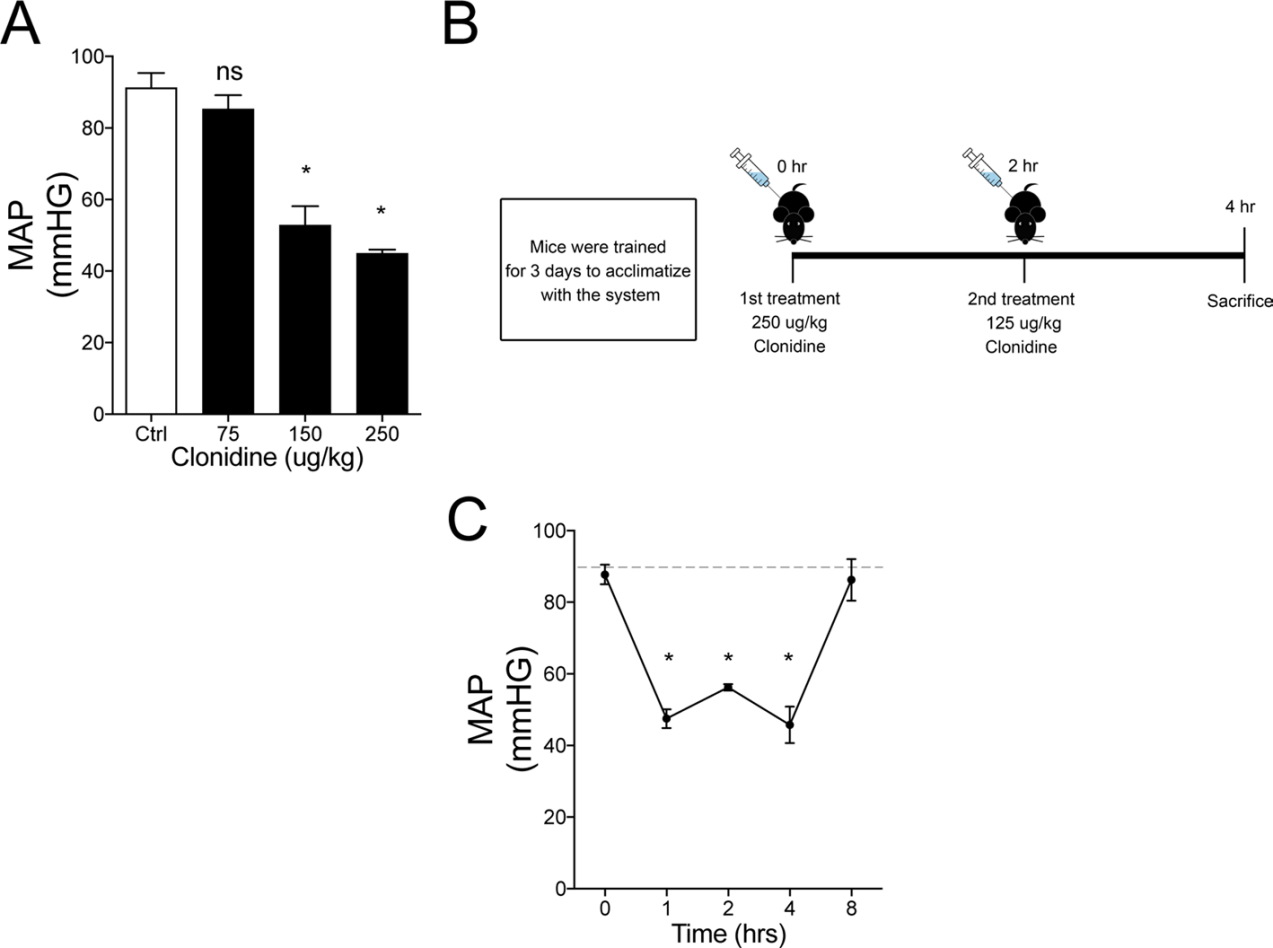
Model of clonidine-induced hypotension in mice. **A** Male C57bl/J6 mice, 10–12 weeks of age, were treated with different clonidine concentrations to identify the concentration sufficient to drop the mean arterial pressure (MAP) by half after 2 h (*p < 0.05, ns = not significant *n* = 3 per group). **B** Schematic representation of the clonidine-induced hypotension protocol used for this study to maintain MAP below 60 mmHg over 4 h. **C** Mice were treated with 250 ug/kg and 125 ug/kg of clonidine at indicated timepoints to maintain a MAP below 60 mmHg for at least 4 h. At 8 h, MAP values were back to normal. (*p < 0.05, ns = not significant, *n* = 4 per group). The dotted lines represent the mean of saline-treated mice across the timepoints

We injected two clonidine (Sigma-Aldrich) doses subcutaneously to induce an experimental shock-like situation in awake 10–12-week-old male C57bl/J6 mice for of 4 h. A duration of 4 h was chosen based on previous kinetic studies of Tie2 mRNA suppression post-experimental sepsis (approximately 90% reduction within 4 h [8]). A minimum of 5 mice per group was used. The first dose of 250 ug/kg at the beginning of the experiment and the second dose of 125 ug/kg 2 h after the first (schematic protocol shown in Fig. 1B). Blood pressure was measured in the tail using non-invasive volume pressure recording (VPR) sensor technology (Coda systems). Before inducing experimental hypotension, mice were first trained for 3 days to limit stress and get them acclimatized with the system. The MAP was calculated as the sum of the systolic blood pressure plus 2 × diastolic blood pressure divided by 3.

After the induction of hypotension for 4 h, mice were anaesthetized with 3.5% isoflurane and maintained with 1.5% isoflurane after which blood was drawn via the retroorbital sinus followed by transcardial perfusion with 10 ml PBS until the lungs, kidney and liver were cleared of blood. Organs were either snap-frozen in liquid nitrogen, filled with 4% PFA for immunochemistry analysis or harvested in buffer solution for EC isolation or measurement of lung wet-to-dry ratio.

### RNA isolation and mRNA expression analysis

Total RNA was extracted from organs snap-frozen in liquid nitrogen and stored at –80 degrees using the RNeasy Mini/Micro Kit (Qiagen, Hilden, Germany) followed by reverse transcription using Transcriptor First Strand cDNA Synthesis (Roche Diagnostics). The RT-qPCR analysis was performed using a LightCycler 480 II (Roche). Gene expression was normalized to the expression of the housekeeping gene Actin, yielding the ∆CT value.

### Immunoblotting

Frozen tissues or isolated ECs were homogenized in radioimmunoprecipitation assay (RIPA) buffer followed by centrifugation at 4 °C for 15 min at 12,000 rpm. The supernatant's protein concentration was determined with the Pierce BCA Protein Assay Kit (Thermo Scientific, Rockland, IL). Proteins were resolved with a 10% polyacrylamide gel electrophoresis then transferred to PVDF (polyvinylidene fluoride) membranes (Merck Millipore, Darmstadt, Germany). The membrane was blocked with 3% bovine serum albumin (BSA) and incubated with a primary antibody overnight (4 °C) followed by incubation with 2nd antibody for 1 h at room temperature. Bands were visualized with SuperSignal West Pico Chemiluminescent Substrate (Life Technologies) and Versa Doc Imaging System Modell 3000 (BioRad). Quantification of immunoblots was done using ImageJ [17]

### Fluorescent immunohistochemistry

Paraffin-embedded Sections (2 µm) from lungs were labelled with primary antibody against Gr-1 (AbD serotec, Puchheim, Germany). Following incubation with secondary antibodies, we used goat anti-rat IgG-HRP (Santa Cruz Biotechnology, CA, USA). For global histomorphologic analysis of lungs, periodic acid-Schiff (PAS) staining was used.

### Wet-to-dry ratio

The lung wet-to-dry (W/D) weight ratio was used as a surrogate of vascular permeability. After inducing hypotension for 4 h, mice were injected subcutaneously with 200 µl of saline (to provide supplementary fluid that can extravasate into tissues that have permeable vessels) and sacrificed under anesthesia; the lungs were carefully removed immediately and were measured (wet weight). The lung tissue was re-weighed after being dried in an oven at 70 °C for 24 h (dry weight). The W/D weight ratio was calculated by dividing the wet by the dry weight.

### Isolation of pulmonary endothelial cells

As previously described [18], the isolation of murine pulmonary ECs was done by enzymatic digestion, followed using anti-CD31-antibody (BD Pharmingen)-conjugated Dynabeads (Life Technologies). After purification, cells were either processed for RNA isolation for mRNA expression analysis or for protein analysis by immunoblotting.

### GATA3 transient transfection in vivo

Following the manufacturer's guidelines, in vivo-jetPEI (Polyplus Transfection) transfection reagent was used as a carrier to overexpress GATA3 in murine pulmonary vasculature transiently. Mice were injected by a single blinded investigator retroorbitally with 40 μg control or GATA3 plasmid DNA (LZRS-GATA3) a kind gift from Dr. Ellen Rothenberg (Addgene plasmid #34836; http://n2t.net/addgene:34836; RRID:Addgene_34836) [19] complexed with in vivo-jetPIE at an N/P ratio of 8 in 200 μl of 5% glucose solution. 24 h after injection, ECs were isolated for RNA and protein expression analysis or hypotension was induced for further studies.

### Conditional GATA3 knockout mice

Eight to 12-week-old mice with inducible endothelial‐specific GATA3 deletion (B6-Gata3^tm1−Jfz^ VE-Cadherin(PAC)-cerERT2) were used [20]. For inducible knockout of the floxed GATA3 alleles in the endothelium, tamoxifen (Sigma-Aldrich, St. Louis, MO) dissolved in corn oil (Sigma-Aldrich) or Miglyol 812 (Caesar & Loretz GmbH) at a 20 mg/mL concentration was injected at 75 mg tamoxifen/kg body weight (BW) intraperitoneally every day for five consecutive days. Age matched littermates treated with Corn oil or Miglyol 812 served as controls. Seven days after the last injection, lungs were either extracted for pulmonary EC isolation, filled with 4% PFA for immunohistochemistry, or harvested to measure lung wet-to-dry ratio.

### Statistical analysis

Results are presented as mean ± SEM unless otherwise specified. Differences between two groups were calculated using non-parametric Mann–Whitney *U* test. For groups of three or more conditions Turkey test was used. A *p* value of less than 0.05 was considered statistically significant Analysis and graphical presentation were performed using GraphPad Prism9 software. Sample size were calculated using G-Power software. The α-error probability was given as 0.05, power as 0.80 and effect size as 2.0.

## Results

### Murine model of persistent hypotension

To study the effect of low microcirculatory flow on endothelial Tie2 regulation in vivo in the absence of an inflammatory milieu, we developed a clonidine induced murine model of prolonged hypotension over 4 h (described in detail in the material section). Clonidine is an approved alpha‐2 adrenoceptor agonist that is used in clinical routine as an antihypertensive drug or as a mild sedative [21]. We found that a dose of 250 ug/kg clonidine was sufficient to reduce the mean arterial pressure (MAP) up to 48% to a level of 45.50 ± 0.8660 mmHg (Fig. 1A). However, since our protocol required the mice to be in a hypotension state for a minimum of 4 h, a re-dosing of 125 ug/kg after 2 h was necessary (Fig. 1B, C). Our clonidine-induced hypotension model also showed an increased concentration in blood urea nitrogen (BUN), glutamic oxaloacetic transaminase (GOT), glutamic pyruvic transaminase (GPT) and lactate dehydrogenase (LDH), indicative of organ dysfunction, a major characteristic of shock (Table 1). All measurements were performed in an automated manner using OLYMPUS AU 400 (Beckman Coulter, Krefeld, Germany).

**Table 1:** Hypotension-induced organ dysfunction

	Saline	Clonidine
LDH, U/L	402.3 ± 29.13 *p* = 0.0003***	1046 ± 64.15
BUN, mmol/L	8.7 ± 0.9 *p* = 0.0357*	11.4 ± 0.5
GPT, U/L	38.4 ± 1.5 *p* = 0.0171*	61.8 ± 5.8
GOT, U/L	154.3 ± 10.3 *p* = 0.0150*	390.3 ± 64.1

*p ≤ 0.05, ***p ≤ 0.001 Unpaired t test with Welch’s correction between groups: Saline vs Clonidine

### Suppression of GATA3 and Tie2 expression by prolonged hypotension

**Fig. 2.**
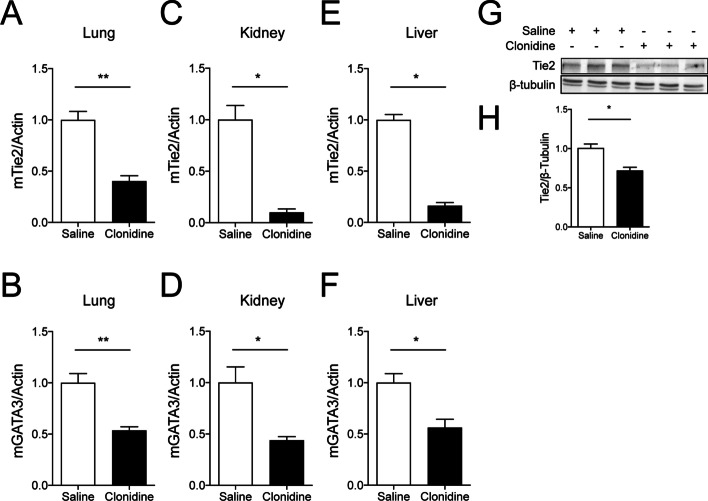
Hypotension diminishes endothelial GATA3 and Tie2 in various organs: Male C57bl/J6 mice, 10–12 weeks of age, were challenged with either saline or clonidine to induce hypotension. **A–B** Lung (**p < 0.01, *n* = 7 per group) (**C**–**D**) kidney (*p < 0.05, *n* = 5 per group) and (**E**–**F**) liver were assessed for Tie2 and GATA3 mRNA level (*p < 0.05, *n* = 5 per group). **G** Representative Immunoblot of whole-lung lysates for Tie2 and β-tubulin expression and (**H**) densitometric quantification of blots (*p < 0.05, *n* = 5 per group)

Having established this model of prolonged hypotension, we sought to study endothelial GATA3 and Tie2 transcripts. We found that both endothelial Tie2 and GATA3 mRNA were reduced in the different vascular beds, such as lung, liver and kidney of hypotensive mice compared to that of the control group (saline-treated mice) (Fig. 2A–F). Upon observing this, we went further to analyze whole lung lysates for total Tie2 protein abundance via immunoblot and found analogous findings (Fig. 2G, H). Of note, other known Tie2 transcription factors, such as ELF-1, NERF2, and Twist1 (Additional file 1: Fig. S1A–C), its canonical ligands Angiopoietin (Angpt)-1 and Angpt-2 (Additional file 2: Fig. S2A–C), including soluble Tie2 (sTie2) (Additional file 3: Fig. S3) were all not affected in our model. These findings confirm our previous in vitro finding that GATA3 might regulate Tie2 in a flow-dependent manner.

### Hypotension-mediated suppression of Tie2 is sufficient to induce vascular permeability

Having established a model that replicates the findings in numerous critical illness models (sepsis, hemorrhagic shock, malaria, anthrax, mesenteric ischemia), we set out to study potential functional effects.

**Fig. 3.**
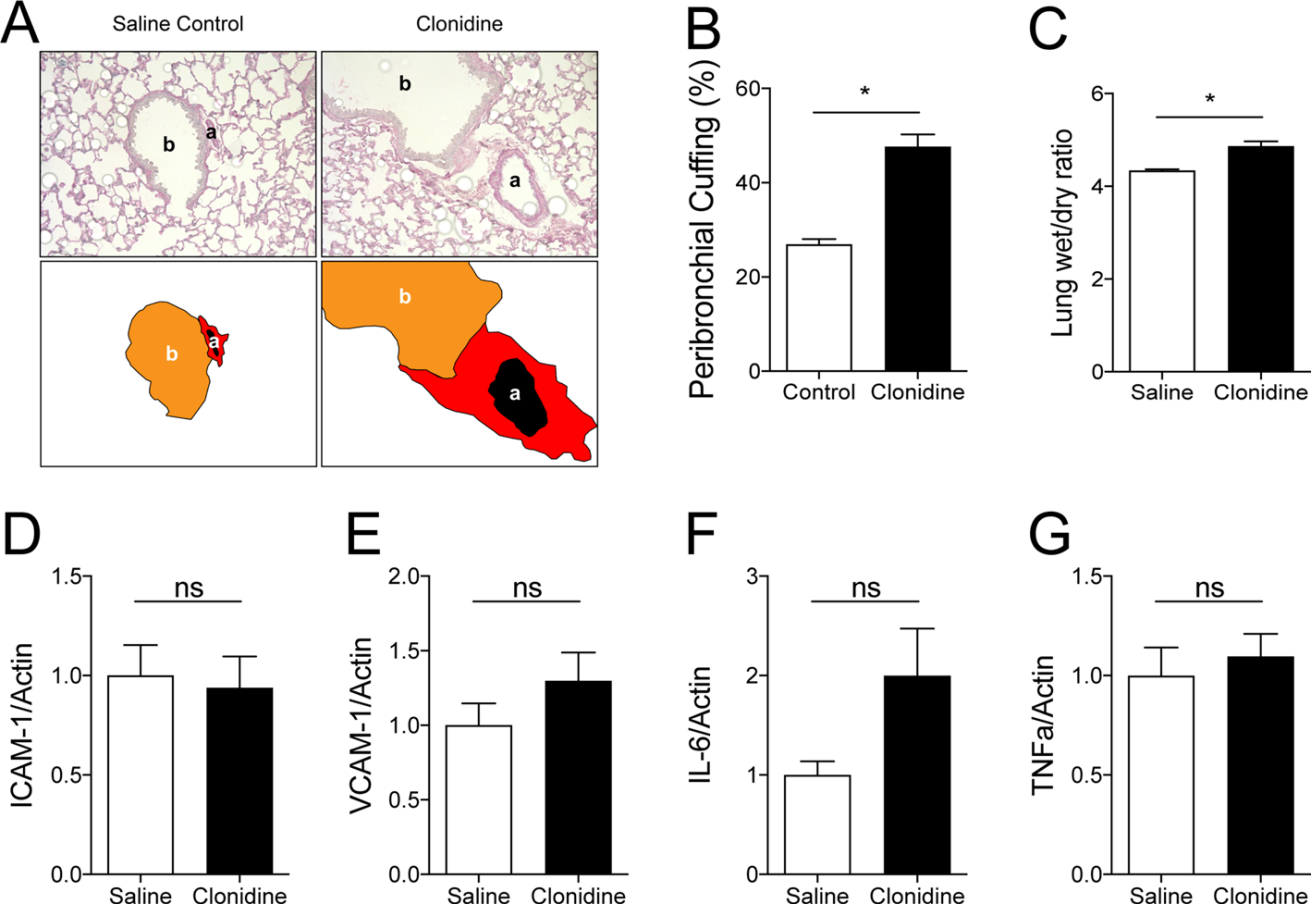
Hypotension-induced loss of GATA3 and Tie2 promotes pulmonary vascular leakage but not inflammation in vivo: **A** Periodic acid-Schiff (PAS) staining of paraffin-embedded lung tissue in clonidine-induced hypotensive mice and saline-treated controls. All images show bronchus (b), their corresponding arteriola (a) and the peribronchial cuffing (a surrogate of lung edema). The schematic illustration below highlights this peribronchial cuff area (in red). **B** Semi-quantification of peribronchial cuffing was performed by systematically surveying whole lung sections. (*p < 0.05, *n* = 5 per group). **C** Lung wet-to-dry weight ratio of saline-treated controls and clonidine-induced hypotension group (*p < 0.05, *n* = 8 per group). Lung mRNA for markers of vascular inflammation and tissue cytokine production (**D**) intercellular adhesion molecule (ICAM)-1, (**E**) vascular cell adhesion molecule (VCAM)-1, (**F**) tumor necrosis factor (TNF)ɑ, and (**G**) interleukin (IL)-6 (ns = not significant, *n* = 5–7 per group)

Consistent with earlier findings in experimental Tie2 reduction on vascular leakage [6], we found that prolonged hypotension was indeed sufficient to induce vascular leakage as indicated by an increased perivascular cuffing (Fig. 3A, B) and an increased lung wet-to-dry ratio (Fig. 3C). However, markers of endothelial inflammation, including adhesion molecule expression, such as intercellular adhesion molecule 1 (ICAM-1) and vascular cell adhesion molecule 1 (VCAM-1) (Fig. 3D, E) as well as cytokine expression (i.e., interleukin (IL)-6, TNFɑ) (Fig. 3F, G) (Additional file 4: Fig. S4), were not different between groups. Consistent with the normal expression of ICAM-1 and VCAM-1, immunofluorescence histology of pulmonary capillaries for Gr1^+^ positive neutrophils revealed no effect of hypotension on tissue infiltration (Additional file 5: Fig. S5A, B). These results indicate that hypotension-induced loss of Tie2 might be sufficient to induce vascular permeability but not inflammation.

### Genetic GATA-3 knockdown reproduces critical findings of the hypotension model

**Fig. 4.**
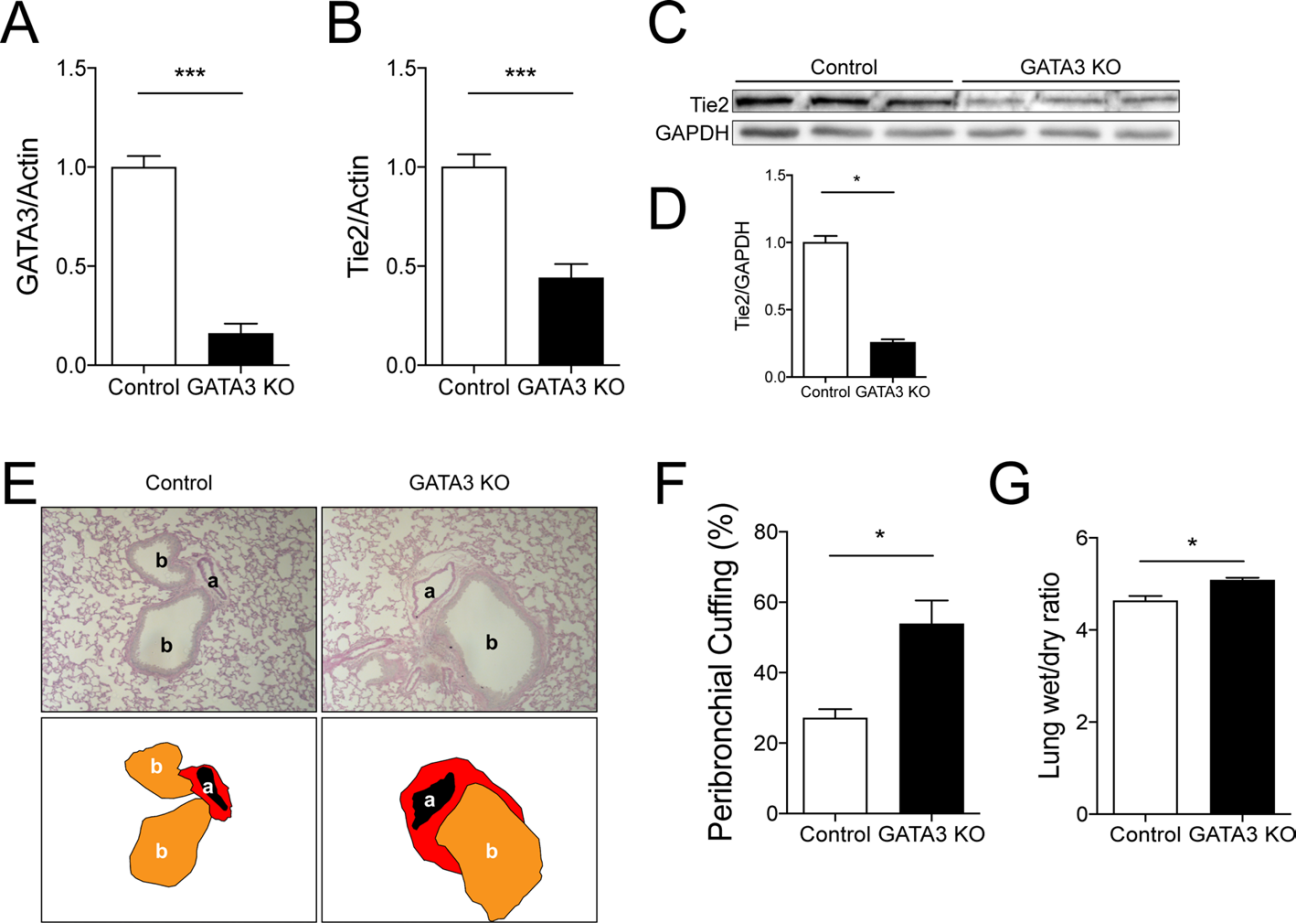
GATA3 regulates Tie2 expression and vascular permeability in vivo. Graph showing (**A**) GATA3 mRNA and (**B**) Tie2 mRNA expression in ECs isolated from the lungs of endothelial GATA3 knock-out mice and controls (***p < 0.001, *n* = 7 per group). **C** Representative immunoblot of whole-lung lysates for Tie2 expression in endothelial GATA3 knock-out mice and control mice (**D**) densitometric quantification of blots (*p < 0.05, *n* = 5 per group). **E** Periodic acid-Schiff (PAS) staining of paraffin-embedded lung tissue in clonidine-induced hypotension mice and saline-treated controls. All images show bronchus (b), their corresponding arteriola (a) and the peribronchial cuffing (a surrogate of lung edema) The schematic illustration below highlights this peribronchial cuff area (in red). **F)** Semi-quantification of peribronchial cuffing was performed by surveying whole lung sections. (*p < 0.05, *n* = 4 per group). **G** Lung wet-to-dry weight ratio of saline-treated controls and clonidine-induced hypotension group (*p < 0.05, *n* = 8 per group)

To determine the role of GATA3 transcription on Tie2 expression in vivo, we used VE-cadherin conditional GATA3 knockout mice. Upon tamoxifen exposure, expression of GATA3 in isolated pulmonary ECs decreased by 84% (Fig. 4A). This genetic drop in GATA3 induced a reduction of Tie2 mRNA by 60% in the pulmonary endothelium (Fig. 4B). Whole lung lysates for total Tie2 abundance via immunoblot confirmed these findings on the protein level (Fig. 4C, D). GATA3–Tie2 attenuation was sufficient to induce spontaneous vascular leakage as indicated by a widespread perivascular cuffing and an increased lung wet-to-dry ratio (Fig. 4E–G). However, although endothelial GATA3 KO had no significant effect on the expression of endothelial adhesion molecules VCAM-1, ICAM-1 (Additional file 6: Fig. S6A, B), pulmonary tissue pro-inflammatory cytokine IL-6 (but not TNFɑ) (Additional file 6: Fig. S6C, D) and pulmonary capillary GR-1^+^ neutrophils (Additional file 6: Fig. S6E, F) were significantly increased, thus indicating that endothelial GATA3 knockout might have off-target effects (with regard to Tie2) inducing endothelial inflammation.

### GATA3 overexpression attenuates Tie2 suppression and vascular leakage

**Fig. 5.**
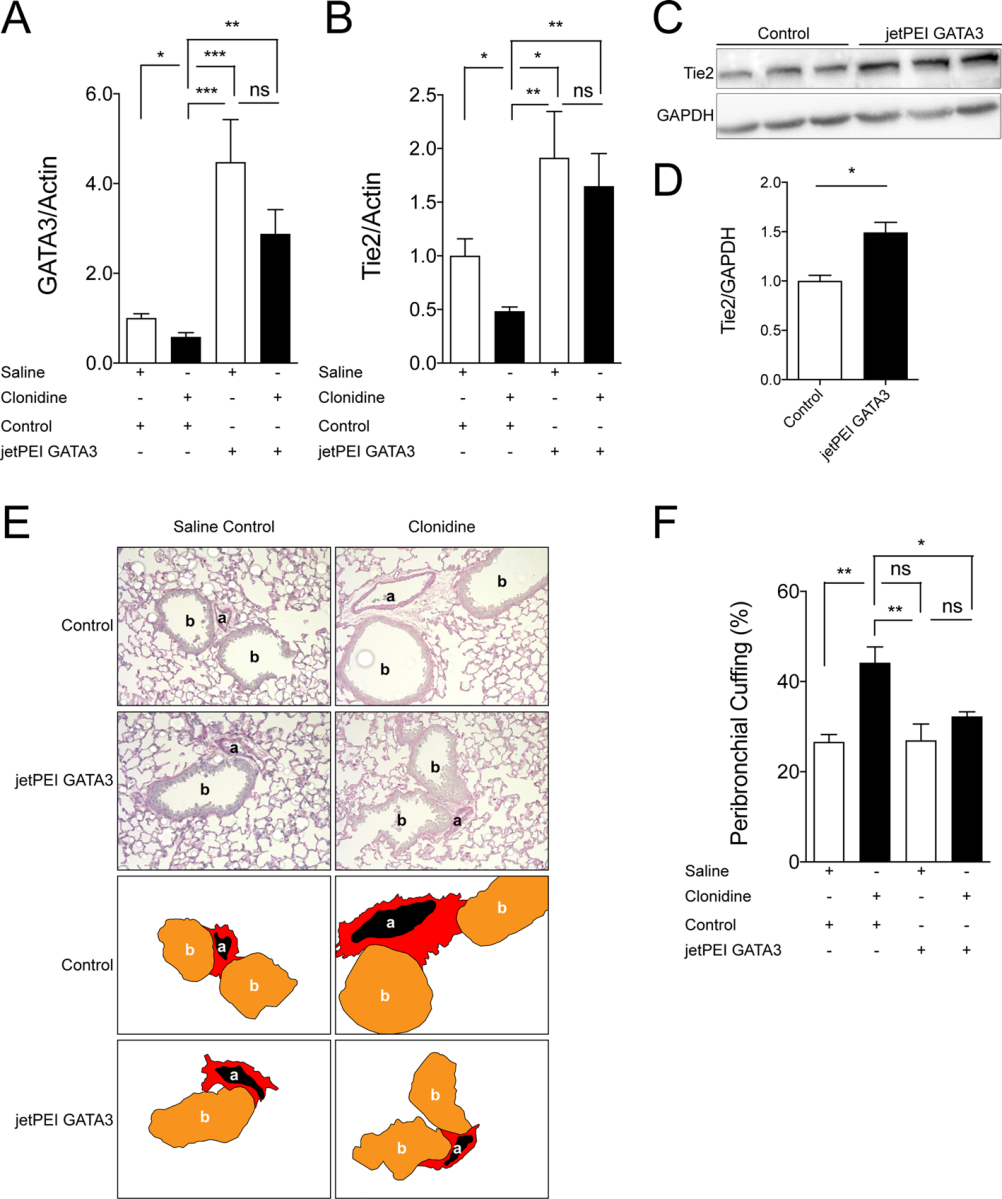
Pulmonary GATA3 overexpression attenuates loss of Tie2, thereby suppressing vascular leakage in vivo. **A** GATA3 mRNA and (**B**) Tie2 mRNA expression in the lungs of GATA3 overexpressing (jetPEI GATA3) and control mice with (clonidine) and without (saline) the induction of hypotension (*p < 0.05, **p < 0.01, ***p < 0.001, ns = not significant, *n* = 6 per group). **C** Representative immunoblot of whole-lung lysates for Tie2 protein expression in GATA3 overexpressing (jetPEI GATA3) and control mice. **D** Densitometric quantification of blots (*p < 0.05, *n* = 4 per group). **E** Periodic acid-Schiff (PAS) staining of paraffin-embedded lung tissue in GATA3 overexpressed and control mice. All images show bronchus (b), their corresponding arteriola (a) and the peribronchial cuffing (a surrogate of lung edema) The schematic illustration below highlights this peribronchial cuff area (in red). **F** Semi-quantification of peribronchial cuffing was performed by surveying whole lung sections. (*p < 0.05, **p < 0.01, ns = not significant. *n* = 4 per group)

From the GATA3 KO results, we inferred that GATA3 overexpression might prevent hypotension-induced loss of Tie2 transcript, thereby opening a potential therapeutic avenue. Hence, we transiently overexpressed GATA3 or control plasmid DNA in mice using a delivery platform termed jetPEI that increased GATA3 mRNA up to × 4 in the lung (Fig. 5A). This increase of GATA3 was sufficient to i) increase baseline Tie2 mRNA expression and ii) to completely protect against the clonidine-induced Tie2 suppression (Fig. 5B). Whole lung lysates for total Tie2 abundance via immunoblot also confirmed the Tie2 increase on the protein level (Fig. 5C, D). On the functional level, GATA3 overexpression was able to attenuate hypotension-induced pulmonary perivascular cuffing (Fig. 5E, F).

## Discussion

**Fig. 6.**
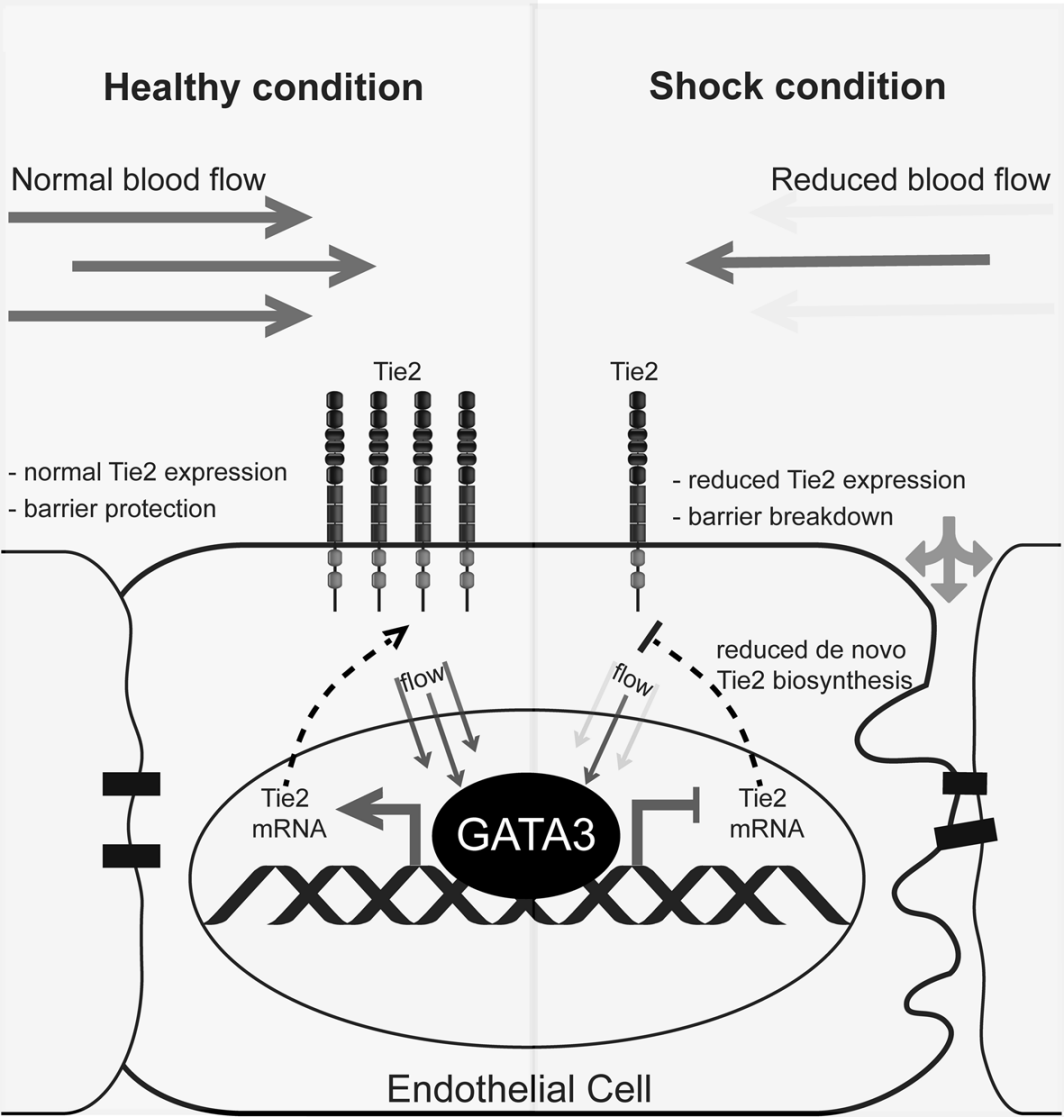
Schematic illustration of the flow-regulated GATA3-dependent attenuation of endothelial Tie2

To our knowledge, we observed for the first time that hypotension is sufficient to induce vascular leakage through the attenuation of the GATA3–Tie2 signaling pathway in vivo. We further confirmed previous studies [8, 16] that GATA3 is indeed a transcriptional regulator of Tie2; using endothelial-specific GATA3 KO mice. As proof of principle, we could show that the transient overexpression of GATA3 is sufficient to completely prevent the hypotension-induced loss of Tie2, thus alleviating vascular leakage. Our findings suggest that the GATA3–Tie2 signaling pathway controls vascular permeability in a flow-dependent manner (Summarized in Fig. 6). Therapeutic interventions targeting this pathway could inhibit vascular permeability in diverse critical illnesses characterized by a low microvascular flow.

Undoubtedly, the role of microvascular flow on endothelial gene responses in the context of critical illnesses is understudied. The finding that diverse critical illnesses have the same effect on Tie2 suppression [5, 6, 9, 22] was striking and initiated a search for common disease features that ultimately led to the hypothesis of a flow-regulated endothelial gene response of Tie2. To clarify this in vitro observation in a translational in vivo approach, we developed a model of persistent hypotension using clonidine which has previously been established to evoke hypotension in mice [23–25] to study Tie2 transcription in the absence of any confounding disease factors, such as inflammation or damage-associated molecular patterns (DAMPS). Notable, clonidine treatment did not induce any significant changes in circulating soluble Tie2 (sTie2) and Angpt2 concentrations when compared to the control group **(**Additional files 2, 3: Figs. S2C, S3), thus eliminating any apparent posttranslational effect of shedding or ligand-mediated deactivation on Tie2 expression and function. That being said, we used clonidine as a model of hypotension to prove our hypothesis in a clean context but one cannot translate it 1 to 1 to a shock scenario associated with a real disease.

Our current data suggest that reduction in mean arterial pressure (MAP) alone is sufficient to reduce endothelial GATA3 and consecutively Tie2 mRNA expression without altering other known Tie2 transcription factors or its ligands (Angpt-1 and Angpt-2). Furthermore, in this regard, it has previously been shown that a drop in blood pressure in a rat model of cardiopulmonary bypass is accompanied by reduced Tie2 expression [26]. The ideal MAP required to protect Tie2 transcription from downregulation is unknown and might even differ inter-individually, potentially influenced by baseline factors associated with the blood pressure under healthy conditions.

The fact that overexpression of GATA3 in murine pulmonary vasculature increased baseline Tie2 and (more importantly) completely block the injurious loss of Tie2 in the hypotension model points towards its therapeutic potential. How could a treatment strategy targeting GATA3 transcription look like at the ICU bedside? Interestingly, GATA3 RNA modulating therapies have been reported to target Th2-cell response in allergic asthma delivered via nasal inhalation [27]. The development of drug-like molecules or antibodies capable of either preventing the loss of or inducing endothelial GATA3 during critical illness, such as sepsis, could represent causative treatment strategy against capillary leak. It is, however, noteworthy to mention that Tie2 regulation during sepsis goes beyond the described transcriptional regulation alone. For example, Tie2 can also be regulated on the posttranscriptional level and by its circulating ligands. A more pragmatic approach might be to combine therapies with the ultimate goal of keeping Tie2 intact and optimizing its net downstream signal.

Our study has some limitations. Although we showed that upon inducing hypotension, GATA3 and Tie2 mRNA expression were reduced in the lung, liver, and kidney, we focused on the lungs for further studies because of the abundant expression of Tie2 in the pulmonary vasculature. From the functional point of view the pulmonary vascular bed is also of highest susceptibility for edema and has often direct life-threatening consequences due the impaired gas exchange. Second, we did not measure lactate or blood gas levels as standard surrogate markers for shock after clonidine treatment. However, we observed an attenuation of pulmonary, renal and liver Kruppel-Like Factor (KLF)-2 transcripts (Additional file 7: Fig. S7A–C). Considering KLF2 is well known to be an endothelial flow-responsive gene [28–30], we presume that flow was indeed regulated in our study. Furthermore, there is a possibility that the relatively high dose of clonidine used to induce and maintain a drop in MAP by approximately 50% might have caused some unwanted side effects (such as impairment in motor coordination, intoxication or bradycardia). However, considering that the blood pressure of the clonidine treated mice was back to baseline level after 4 h of completing the experiment and the pulmonary ECs of the clonidine treated mice had similar VCAM-1, ICAM-1, TNFα mRNA expressions in addition to similar concentration of circulating serum TNFα compared to control mice suggests that whatever effect clonidine might have was minor or temporary with no significant influence on outcome parameters. In addition, we only used peribronchial cuffing (a surrogate of lung edema) as a measure of vascular permeability upon GATA3 overexpression. Additional experiments to support our positive findings would be desirable.

## Conclusions

Our data show that hypotension observed in many critical illnesses may lead to an attenuation of flow-induced endothelial GATA3 transcription, thus leading to an acute loss of Tie2 expression, which might provoke vascular permeability. Hence, designing therapeutic strategies to prevent hypotension-induced GATA3 downregulation might help ameliorate vascular leakage in diverse critical illnesses.

## Supplementary Information

**Additional file 1: Figure S1.** Flow-regulation of other known transcription regulators of Tie2 in vivo: Male C57bl/J6 mice, 10–12 weeks of age, were challenged with either saline or clonidine to induce hypotension. (**A**) ELF1, (**B**) NERF1 and (**C**) TWIST1 mRNA expression levels in the lungs were assessed (ns = not significant n = 5 per group).

**Additional file 2: Figure S2.** Effect of experimental hypotension on the expression of Tie2 ligands. Male C57bl/J6 mice, 10–12 weeks of age, were challenged with saline or clonidine to induce hypotension. (**A**) Angiopoietin 1 mRNA (ns = not significant n = 5 per group) (**B**) Angiopoietin 2 mRNA expression levels in the lungs were assessed (ns = not significant n = 5 per group). (**C**) Serum Angiopoietin 2 concentration (ns = not significant n = 5 per group).

**Additional file 3: Figure S3.** Effect of experimental hypotension on Tie2 cleavage. Male C57bl/J6 mice, 10–12 weeks of age, were challenged with saline or clonidine to induce hypotension after which the concentration of circulating soluble Tie2 was quantified via ELISA. (ns = not significant n = 5 per group).

**Additional file 4: Figure S4.** Effect of experimental hypotension on the proinflammatory cytokine, TNFα. Male C57bl/J6 mice, 10–12 weeks of age, were challenged with saline or clonidine to induce hypotension after which the serum concentration of TNFα was quantified via ELISA. (ns = not significant n = 5 per group).

**Additional file 5: Figure S5.** Neutrophil tissue infiltration: (**A**) Representative lung immunostaining of granulocyte differentiation antigen (Gr) − 1 (red) (nuclear staining with 4′,6-diamidino-2-phenylindole (blue), autofluorescence is shown in green, in clonidine induced hypotension group vs saline treated group (**B**) Semi quantification of whole lung cross sections by evaluating Gr-1^+^ cells per field of vision (ns = not significant n = 5 per group).

**Additional file 6: Figure S6.** Effect of endothelial GATA3 knockdown on inflammation: (**A**) intercellular adhesion molecule (ICAM)-1, (**B**) vascular cell adhesion molecule (VCAM)-1, (**C**) interleukin (IL)6 and (**D**) tumor necrosis factor (TNF)ɑ mRNA expression in lung were assessed (*p < 0.05, n = 7 per group). (**E**) Representative lung immunostaining of granulocyte differentiation antigen (Gr) − 1 (red) (nuclear staining with 4′,6-diamidino-2-phenylindole, (blue), autofluorescence is shown in green, in VE-Cad-GATA3 KO group and control group (**F**) Semi quantification of whole lung cross sections by evaluating Gr-1 + cells per field of vision (*p < 0.05, n = 5 per group).

**Additional file 7: Figure S7**. Hypotension suppresses endothelial KLF2 mRNA expression in various organs indicating reduced flow in different vascular beds: Male C57bl/J6 mice, 10–12 weeks of age, were challenged with either saline or clonidine to induce hypotension. (**A**) The lung (**p < 0.01, n = 7 per group) (**B**) kidney (*p < 0.05, n = 4 per group) and (**C**) liver were assessed for KLF2 mRNA level (*p < 0.05, n = 4 per group).

## Data Availability

All data sets generated for this study are included in the article and Additional files.

## Abbreviations

Angpt-1: Angiopoietin 1
Angpt-2: Angiopoietin 2
BUN: Blood urea nitrogen
ECs: Endothelial cells
GATA3: GATA Binding Protein 3
GATA4: GATA Binding Protein 4
GOT: Glutamic oxaloacetic transaminase
GPT: Glutamic pyruvic transaminase
ICAM-1: Intercellular Adhesion Molecule 1
ICU: Intensive Care Unit
IL-6: Interleukin 6
KLF2: Kruppel-Like Factor 2
LDH: Lactate dehydrogenase
MAP: Mean Arterial Pressure
*NF-κB*: Nuclear Factor kappa-light-chain-enhancer of activated B cells
PFA: Paraformaldehyde
RIPA: Radioimmunoprecipitation assay buffer
TNFɑ: Tumor necrosis factor alpha
Twist1: Twist Family BHLH Transcription Factor 1
VCAM-1: Vascular Cell Adhesion Molecule 1
W/D: Wet-to-dry
