# Supplementary figures and images for: Flow-dependent regulation of endothelial Tie2 by GATA3 in vivo
Source: Intensive Care Med Exp. 2021 Aug 2;9:38. doi: 10.1186/s40635-021-00402-x (PMC8326239; doi:10.1186/s40635-021-00402-x)

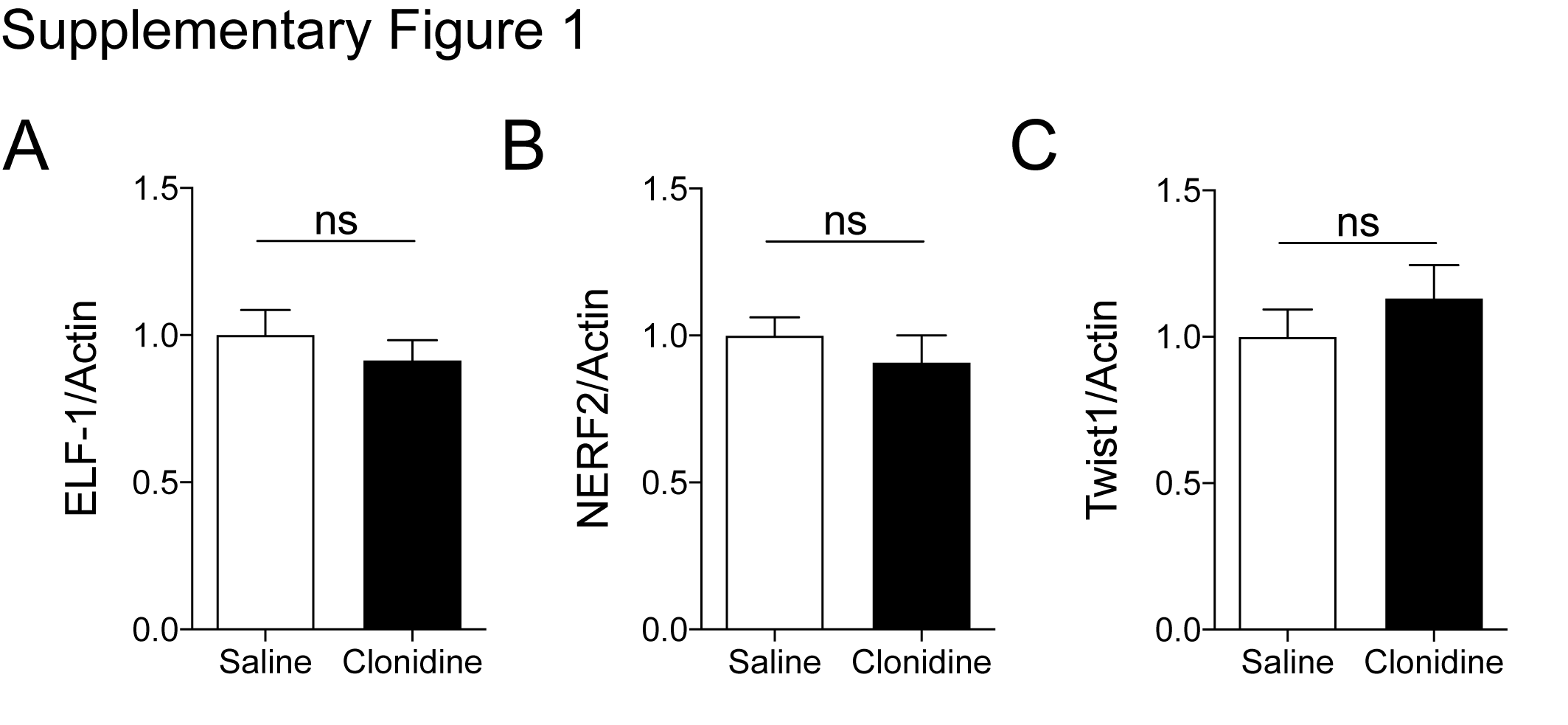

Supplement: Supplementary file 1 — Additional file 1: Figure S1. Flow-regulation of other known transcription regulators of Tie2 in vivo: Male C57bl/J6 mice, 10–12 weeks of age, were challenged with either saline or clonidine to induce hypotension. (A) ELF1, (B) NERF1 and (C) TWIST1 mRNA expression levels in the lungs were assessed (ns = not significant n = 5 per group). [file 40635_2021_402_MOESM1_ESM.tif]

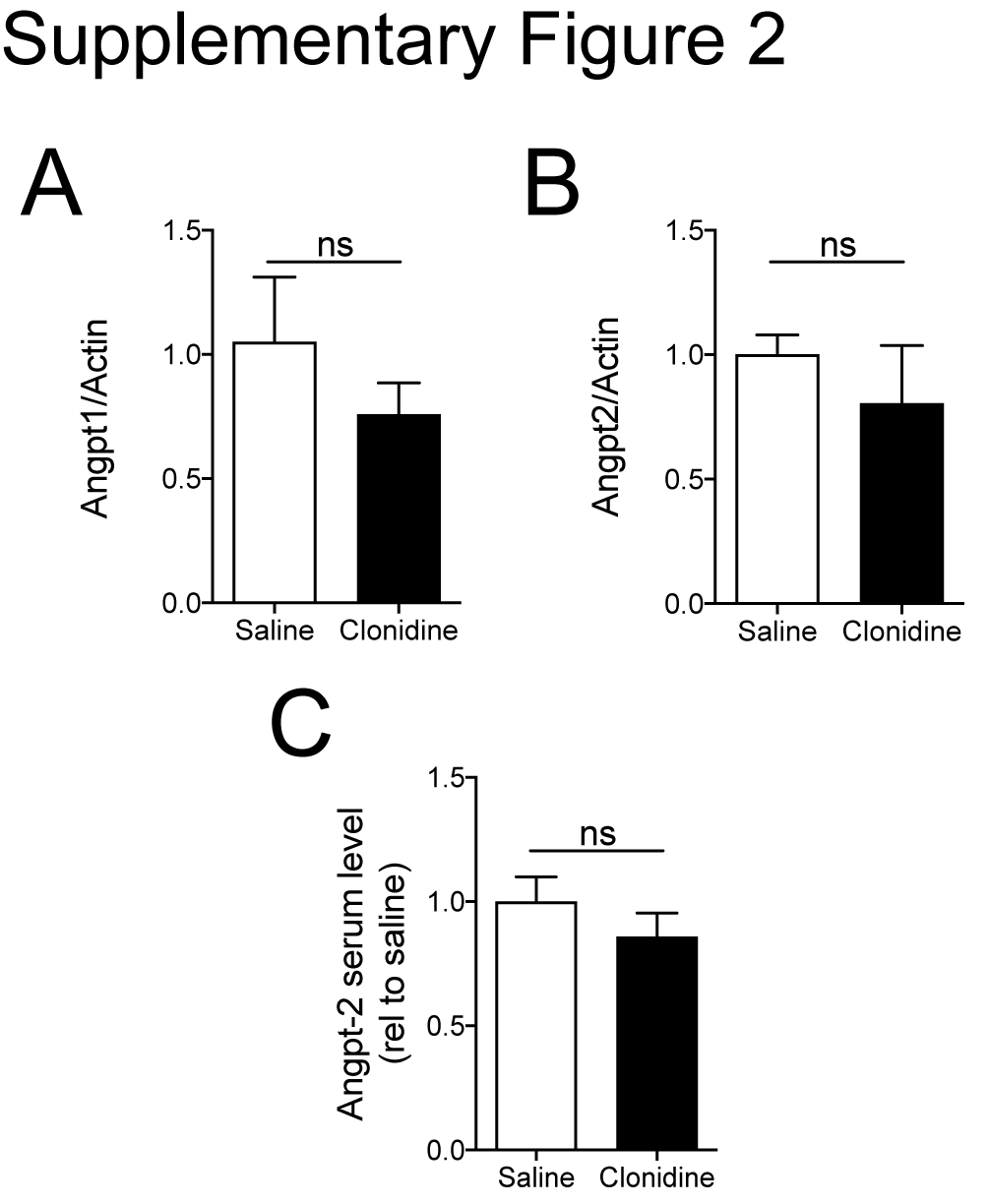

Supplement: Supplementary file 2 — Additional file 2: Figure S2. Effect of experimental hypotension on the expression of Tie2 ligands. Male C57bl/J6 mice, 10–12 weeks of age, were challenged with saline or clonidine to induce hypotension. (A) Angiopoietin 1 mRNA (ns = not significant n = 5 per group) (B) Angiopoietin 2 mRNA expression levels in the lungs were assessed (ns = not significant n = 5 per group). (C) Serum Angiopoietin 2 concentration (ns = not significant n = 5 per group). [file 40635_2021_402_MOESM2_ESM.tif]

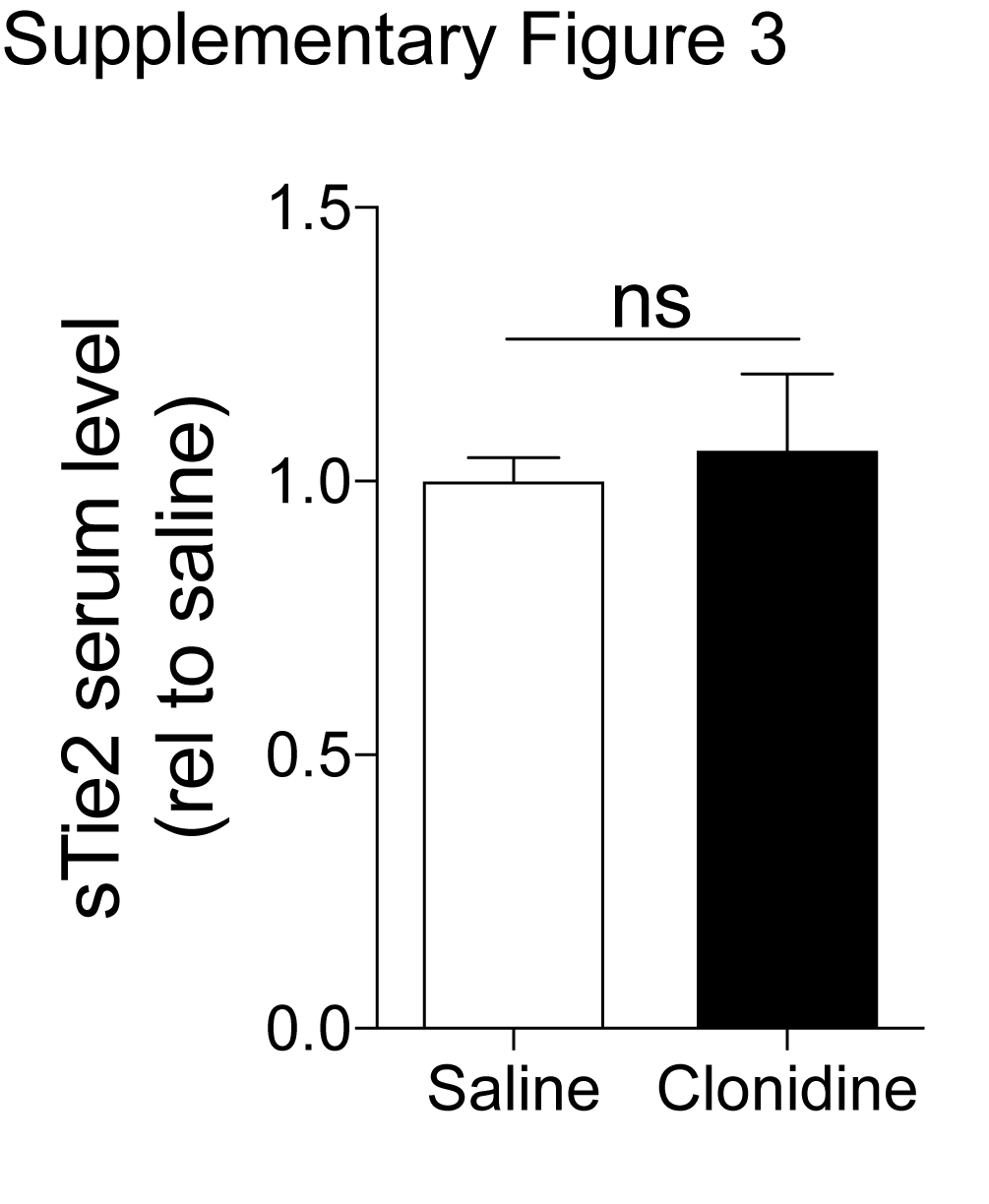

Supplement: Supplementary file 3 — Additional file 3: Figure S3. Effect of experimental hypotension on Tie2 cleavage. Male C57bl/J6 mice, 10–12 weeks of age, were challenged with saline or clonidine to induce hypotension after which the concentration of circulating soluble Tie2 was quantified via ELISA. (ns = not significant n = 5 per group). [file 40635_2021_402_MOESM3_ESM.tif]

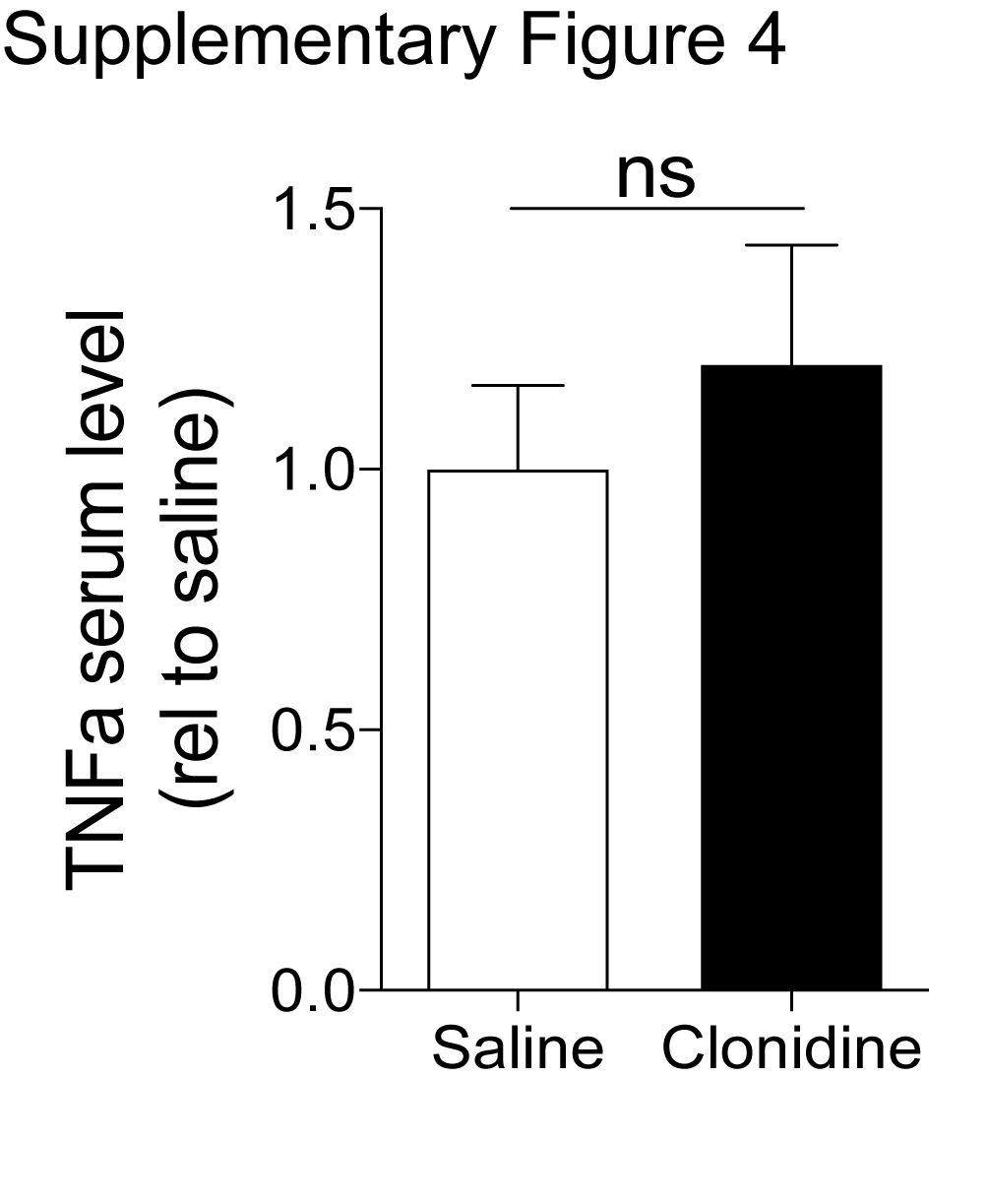

Supplement: Supplementary file 4 — Additional file 4: Figure S4. Effect of experimental hypotension on the proinflammatory cytokine, TNFα. Male C57bl/J6 mice, 10–12 weeks of age, were challenged with saline or clonidine to induce hypotension after which the serum concentration of TNFα was quantified via ELISA. (ns = not significant n = 5 per group). [file 40635_2021_402_MOESM4_ESM.tif]

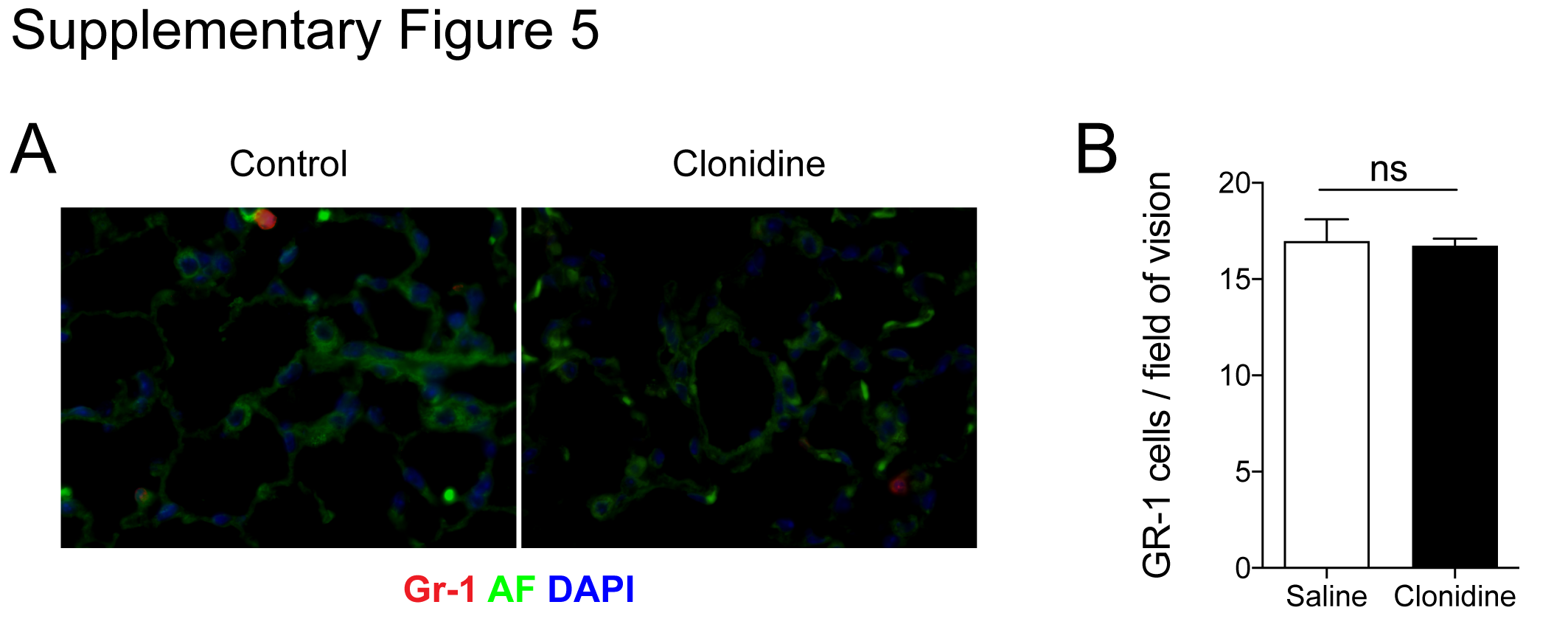

Supplement: Supplementary file 5 — Additional file 5: Figure S5. Neutrophil tissue infiltration: (A) Representative lung immunostaining of granulocyte differentiation antigen (Gr) − 1 (red) (nuclear staining with 4′,6-diamidino-2-phenylindole (blue), autofluorescence is shown in green, in clonidine induced hypotension group vs saline treated group (B) Semi quantification of whole lung cross sections by evaluating Gr-1+ cells per field of vision (ns = not significant n = 5 per group). [file 40635_2021_402_MOESM5_ESM.tif]

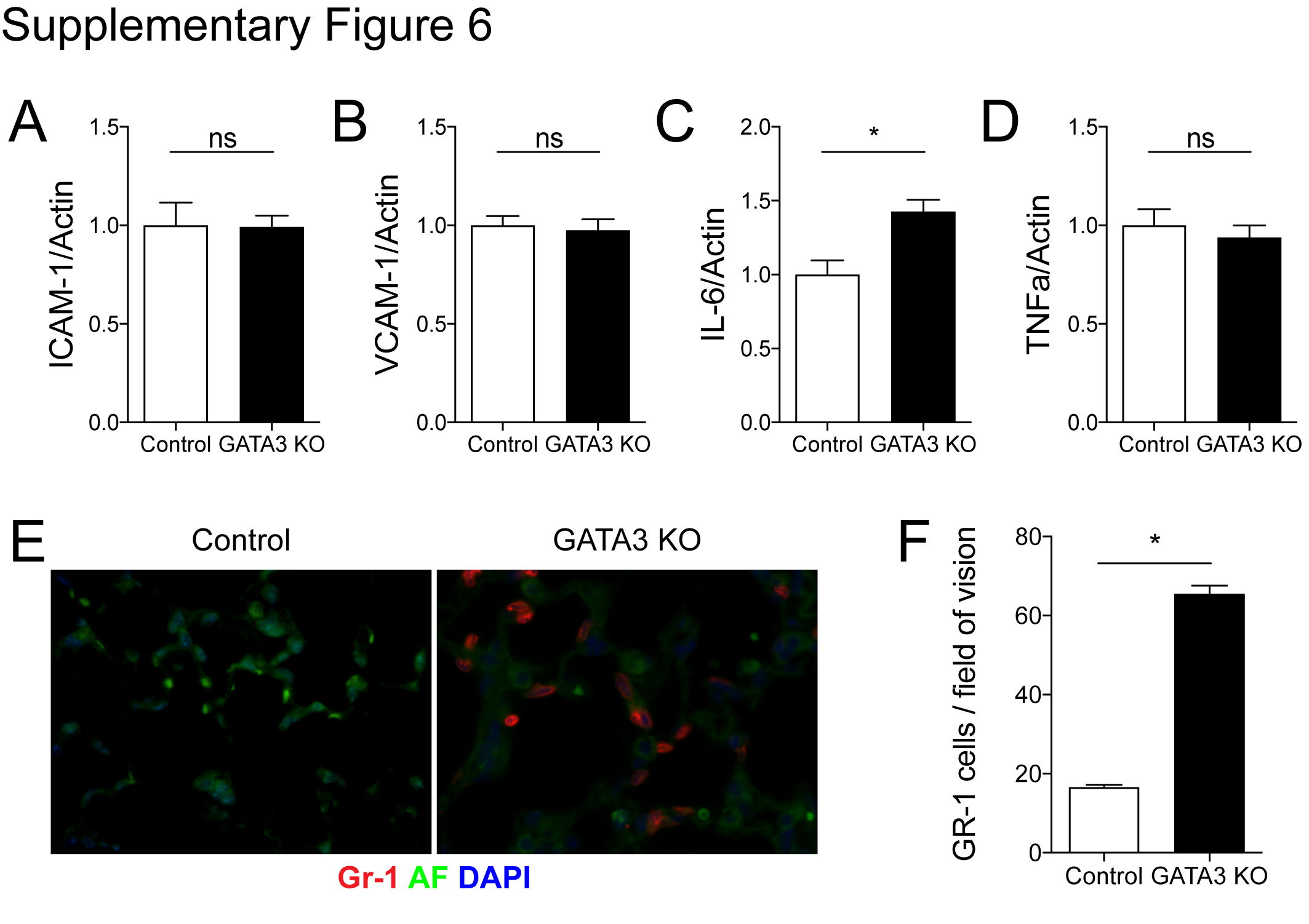

Supplement: Supplementary file 6 — Additional file 6: Figure S6. Effect of endothelial GATA3 knockdown on inflammation: (A) intercellular adhesion molecule (ICAM)-1, (B) vascular cell adhesion molecule (VCAM)-1, (C) interleukin (IL)6 and (D) tumor necrosis factor (TNF)ɑ mRNA expression in lung were assessed (*p < 0.05, n = 7 per group). (E) Representative lung immunostaining of granulocyte differentiation antigen (Gr) − 1 (red) (nuclear staining with 4′,6-diamidino-2-phenylindole, (blue), autofluorescence is shown in green, in VE-Cad-GATA3 KO group and control group (F) Semi quantification of whole lung cross sections by evaluating Gr-1 + cells per field of vision (*p < 0.05, n = 5 per group). [file 40635_2021_402_MOESM6_ESM.tif]

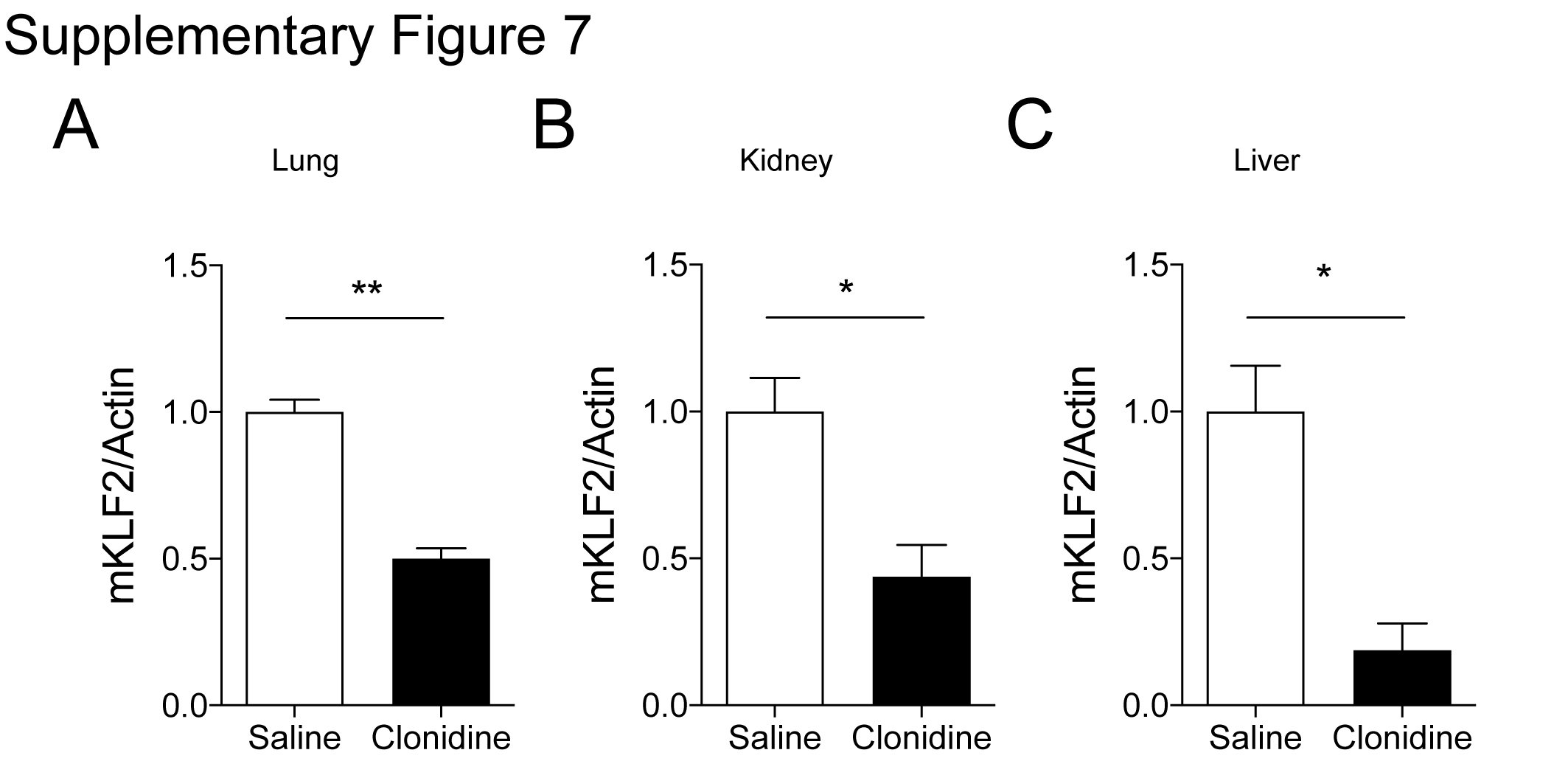

Supplement: Supplementary file 7 — Additional file 7: Figure S7. Hypotension suppresses endothelial KLF2 mRNA expression in various organs indicating reduced flow in different vascular beds: Male C57bl/J6 mice, 10–12 weeks of age, were challenged with either saline or clonidine to induce hypotension. (A) The lung (**p < 0.01, n = 7 per group) (B) kidney (*p < 0.05, n = 4 per group) and (C) liver were assessed for KLF2 mRNA level (*p < 0.05, n = 4 per group). [file 40635_2021_402_MOESM7_ESM.tif]
